# A partial T-DNA insertion near *KNAT1* results in lobed *Arabidopsis* *thaliana* leaves

**DOI:** 10.17912/micropub.biology.000253

**Published:** 2020-05-21

**Authors:** Karah Moulton, Stephanie Diaz, Ashley Strother, C. Nathan Hancock

**Affiliations:** 1 Department of Biology and Geology, University of South Carolina Aiken, Aiken, SC; 2 Department of Biochemistry, Purdue University, West Lafayette, IN; 3 Department of Pathology, University of Texas Medical Branch, Galveston, TX

**Figure 1:**
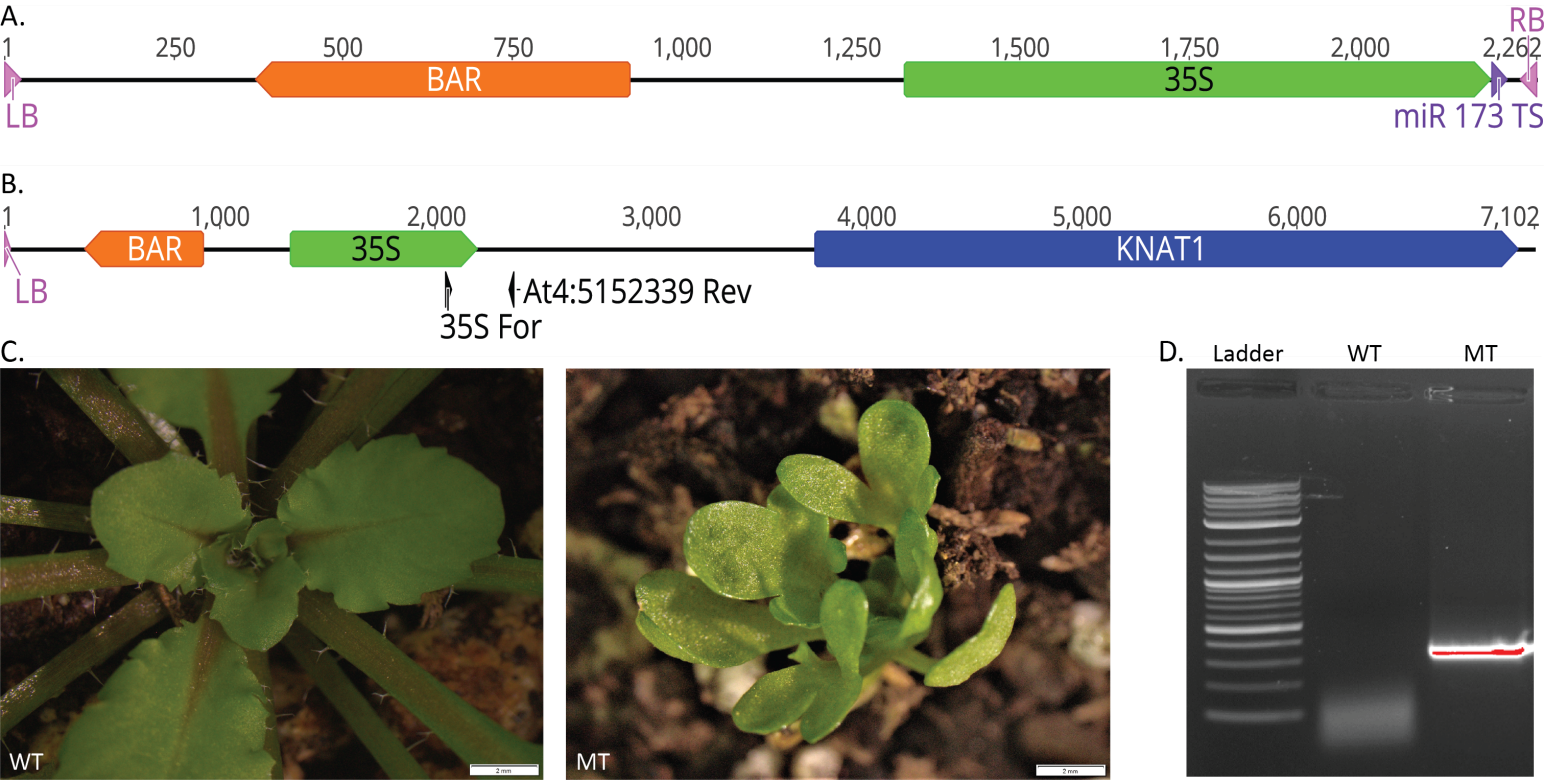
**Generation of the Gamora** **Mutant** (A) Diagram of the MIGS T-DNA composed of left border (LB), Basta herbicide resistance (BAR) gene, the CaMV 35S promoter (35S), the miR173 target sequence, and the right border (RB). (B) Diagram of the T-DNA insertion directly upstream of *KNAT1* (*At4G08150*) in the Gamora mutant. Location of the 35S For and At4:5152339 Rev primers used for the PCR are shown as black arrows. Note that part of the miR173 target sequence and the entire right border are missing. Gene diagrams were made using Geneious version 2020.0 created by Biomatters. (C) Comparison of wild-type *Arabidopsis thaliana* (WT) and the Gamora mutant phenotype (MT) at 6 weeks of development. (D) PCR results from amplification of wild-type *Arabidopsis* (WT) and Gamora mutant (MT) DNA with 35S For and At4:5152339 Rev primers. The 600 bp band in the MT lane indicates that the Gamora mutant contains the T-DNA as shown in B.

## Description

T-DNA tagging is a method to generate mutations in plants by random insertion. It is an important tool for the study of gene function in *Arabidopsis* because it allows you to see how the plant responds when expression of a specific protein is altered. T-DNAs carrying promoter elements that can cause transcriptional activation, called activation tags, have previously been shown to be effective at identifying novel genes (Tani *et al.* 2004). We attempted to develop a gene silencing T-DNA, by modifying the existing microRNA-induced gene silencing (MIGS) platform (Han *et al.* 2015). In this platform, the transgene consists of a promoter driving expression of a miR173 target sequence directly adjacent to the gene sequence to be silenced (Zhang 2014). Expression produces an mRNA which is bound by the complementary, naturally occurring *Arabidopsis* microRNA, miR173. This induces the production of small interfering RNAs (siRNAs) which target homologous transcripts for degradation (Zhang 2014).

The T-DNA transgene containing the BAR gene, a CaMV 35S promoter, and the miR173 target sequence was made by modifying the existing pMIGS vector (Han *et al.* 2015) (Fig. 1A). The T-DNA transgene was transformed into wild-type *Arabidopsis* *thaliana* plants using the floral dip method (Clough *et al.* 1998). Screening for mutant phenotypes in the offspring, we identified one plant, named Gamora, which exhibited altered leaf shape, delayed flowering, and a reduced seed set in a dominant manner (Fig. 1C). Whole genome sequencing of the Gamora mutant using Oxford Nanopore MinION sequencing (Michael *et al.* 2018) produced 111,593 reads with an average length of 6 kb, resulting in approximately 670 Mb of sequence. One read contained the T-DNA sequence along with adjacent *Arabidopsis* genome sequence, allowing us to determine where the transgene had inserted in the genome (Fig. 1B). The T-DNA is inserted upstream of *KNAT1* (*At4G08150*), likely in the promoter region. PCR amplification (Fig. 1D) and sequencing of the transgene/genome junction site revealed that the T-DNA right border and part of the miR173 target sequence were missing, but the CaMV 35S promoter was still intact (Fig. 1B). Lacking these sequences, the T-DNA is likely to function as an activation tag because the CaMV 35S promoter has been shown to induce the expression of nearby genes (Odell *et al.* 1985). *KNAT1* has been identified previously and is known to encode for a KN1-like homeodomain protein which is primarily localized in the shoot apical meristem in *Arabidopsis* (Lincoln *et al.* 1994). Overexpression of the maize homolog for *KNAT1* (*Kn1*) under control of the 35S promoter in tomato resulted in dwarfed and bushy plants (Hareven *et al.* 1996). It has also been shown that overexpression of *Kn1* and *KNAT1* under control of the 35S promoterin *Arabidopsis* results in plants with highly lobed leaves, defects in floral development, reduced fertility, slow growth, and dominant inheritance, similar to the Gamora phenotype (Lincoln *et al.* 1994). Together, this suggests that the Gamora phenotype is due to overexpression of the *KNAT1* gene.

The results obtained from this project provide insight into the development and use of *Arabidopsis* gene discovery tools. Though this study identified a mutant phenotype and its underlying gene, it also suggests that there are limitations to our MIGS-based silencing tagging design. Our strategy requires that the miR173 target sequence be placed near the ends of the T-DNA, which increases the probability for loss of this sequence upon insertion. Afolabi *et al.* (2004) found that non-intact T-DNAs were present in over 70% of transgenic rice lines, in most cases reflecting loss of the mid to right border portion of the T-DNA. This suggests that we may have better success with integration of the miR173 target sequence if we include more sequence between the target sequence and the right border. Our study also indicates that Nanopore sequencing can be successfully utilized to identify transgene locations in *Arabidopsis*.

## Methods

**MIGS Transgene Design and Plant Transformation**

The pEG100 MIGS T plasmid was produced by transferring the *EcoR*I to *Xba*I fragment from pMIGS (Han *et al.* 2015) to pEarleyGate100 (Earley *et al.* 2006). The resulting plasmid was then digested with *Pme*I and *Xba*l, blunted with T4 polymerase, and ligated to delete the region between the target site and the right border. Wild-type *Arabidopsis thaliana* plants were transformed using the floral dip method as described by Clough *et al.*, 1998. The transgenic offspring were selected using Basta herbicide.

**Oxford Nanopore MinION sequencing**

DNA was extracted from 15 pooled mutant plants using the CTAB method (Liu *et al.* 1995) and then purified using the E.Z.N.A Plant DNA purification kit (Omega Bio-tek, Norcross, GA). The DNA library was prepared with the ONT Ligation Sequencing Kit 1D (Oxford Nanopore Technologies, Oxford Science Park, UK) by the Functional Genomics Core at the University of South Carolina according to the recommended protocol (Michael *et al.* 2018). The library was sequenced using the Nanopore R9.4 Spot-On Flow cell for 24 hours. Geneious Software was used to BLAST the reads against the transgene sequence.

**PCR**

PCR analysis was performed on wild-type *Arabidopsis* (WT) and Gamora mutant (MT) DNA with 2X Taq RED Maser Mix, 1.5 mM MgCl_2_, and 35S For (AGACGTTCCAACCACGTCTTCAAAGCAAG) and At4:5152339 Rev (TGCATTCGAAATGTTTTCTTTTCC) primers flanking the region between the CaMV 35S promoter and *AtG08151* (Fig. 1B). The PCR reaction was a 10 μl reaction that included a 4 min denaturation at 95°C, then 30 cycles of (30 sec., denaturation at 95°C, 30 sec annealing at 58°C, and a 1 min 30 sec extension at 72°C) and a final extension time of 7 min at 72°C.
